# Complete Genome Sequence of Adlercreutzia equolifaciens subsp. *celatus* DSM 18785

**DOI:** 10.1128/MRA.00354-21

**Published:** 2021-05-13

**Authors:** Haruno Takahashi, Jiayue Yang, Hiromitsu Yamamoto, Shinji Fukuda, Kazuharu Arakawa

**Affiliations:** a Institute for Advanced Biosciences, Keio University, Tsuruoka, Yamagata, Japan; b Systems Biology Program, Graduate School of Media and Governance, Keio University, Fujisawa, Kanagawa, Japan; c Intestinal Microbiota Project, Kanagawa Institute of Industrial Science and Technology, Kawasaki, Kanagawa, Japan; d Transborder Medical Research Center, University of Tsukuba, Tsukuba, Ibaraki, Japan; e Faculty of Environment and Information Studies, Keio University, Fujisawa, Kanagawa, Japan; f Exploratory Research Center on Life and Living Systems, National Institutes of Natural Sciences, Myodaiji, Okazaki, Japan; University of Rochester School of Medicine and Dentistry

## Abstract

Adlercreutzia equolifaciens subsp. *celatus* DSM 18785 was isolated from the cecal contents of a rat and is an obligately anaerobic equol-producing bacterium. Here, we report the finished and annotated genome sequence of this organism, which has a genome size of 2,929,991 bp and a G+C content of 63.2%.

## ANNOUNCEMENT

Adlercreutzia equolifaciens subsp. *celatus* DSM 18785 (Asaccharobacter celatus DSM 18785) is a Gram-positive, non-spore-forming, and obligately anaerobic bacterium which has been isolated from the cecal contents of a rat in Japan (1). Strain DSM 18785 produces equol from the soy isoflavone daidzein, which is an agonist of the female hormone estrogen (1). The estrogen-like action of soy isoflavones and their metabolites has long been thought to have health benefits. Equol is one of the metabolites of soy isoflavones converted by gut microbes. Recently, it has been the focus of research due to its physiological action (2, 3).

In this study, strain DSM 18785 was obtained from the Japan Collection of Microorganisms (JCM), RIKEN BRC, which participates in the National BioResource Project of the MEXT, Japan. A single colony was cultured overnight at 37°C and grown in Gifu anaerobic medium (GAM) broth (Nissui) supplemented with 0.5% arginine (adjusted to a pH of 7 by 1 N HCl) (4). Genomic DNA was extracted and purified using a Genomic-tip 20/G kit (Qiagen) according to the manufacturer’s protocol. A long-read sequencing library was prepared using a rapid barcoding sequencing kit (product number SQK-RBK004; Oxford Nanopore Technologies) and sequenced using a FLO-MIN106 flow cell on a GridION device (Oxford Nanopore Technologies). The reads were base called, demultiplexed, and adapter trimmed using GridION v.20.06.17 software with Guppy high-accuracy mode.

From a total of 728.4 Mb long reads (*N*_50_, 13.4 kbp) sequenced, reads over 20 kbp (287 Mbp in total, for an estimated coverage of 100×) were used for assembly with Canu v.2.1.1 (5). The reads were not filtered by quality nor error corrected prior to assembly. The genome was assembled into a single contig and was circularized manually by deleting the overlapping end. The draft assembly was subsequently error corrected with one round of Pilon v.1.23 (6), polished using 59.9 million raw Illumina short reads obtained from Sequence Read Archive (SRA) data (accession number SRX5082739) (7) in the National Center for Biotechnology Information (NCBI) (8) mapped to the Canu assembly using the Burrows-Wheeler Aligner (BWA) v.0.7.11 (9). The genome completeness was assessed using the CheckM tool provided by the DDBJ Fast Annotation and Submission Tool (DFAST) (10, 11), resulting in 100% completeness with the *Coriobacteriaceae* taxon. The genes were annotated using DFAST, and the genome was rotated according to the location of the *dnaA* gene. The final genome size is 2,929,991 bp, with a G+C content of 63.2%, containing 2,466 putative coding sequences (CDSs), 9 rRNAs, and 51 tRNAs. Default parameters were used for all software unless otherwise specified.

As previous reported, the equol production genes daidzein reductase (*dzr*), dihydrodaidzein reductase (*ddr*), tetrahydrodaidzein reductase (*tdr*), and dihydrodaidzein racemase which present in *A. equolifaciens* subsp. *equolifaciens* DSM 19450^T^ (12) were also found in *A. equolifaciens* subsp. celatus DSM 18785 (13). The homologs of two giant genes, AEQU_0093 and AEQU_1251, encoding putative extracellular surface proteins in *A. equolifaciens* subsp. *equolifaciens* DSM 19450^T^ (14) were found in *A. equolifaciens* subsp. *celatus* DSM 18785 as well. However, the sizes of the open reading frames were smaller, 37,689 bp and 61,890 bp, respectively. The complete genome sequence reported in this study will enable comparative analysis with closely related species to elucidate the mechanisms allowing the production of equol.

### Data availability.

The chromosome sequence reported here was deposited in the DDBJ under accession number AP024470, and the raw reads were deposited in the Sequence Read Archive (SRA) under BioProject accession number PRJNA698616.

## References

[1] Minamida K, Ota K, Nishimukai M, Tanaka M, Abe A, Sone T, Tomita F, Hara H, Asano K. 2008. *Asaccharobacter celatus* gen. nov., sp. nov., isolated from rat caecum. Int J Syst Evol Microbiol 58:1238–1240. doi:10.1099/ijs.0.64894-0. 18450720

[2] Setchell KDR, Brown NM, Lydeking-Olsen E. 2002. The clinical importance of the metabolite equol—a clue to the effectiveness of soy and its isoflavones. J Nutr 132:3577–3584. doi:10.1093/jn/132.12.3577. 12468591

[3] Yuan J-P, Wang J-H, Liu X. 2007. Metabolism of dietary soy isoflavones to equol by human intestinal microflora—implications for health. Mol Nutr Food Res 51:765–781. doi:10.1002/mnfr.200600262. 17579894

[4] Gotoh A, Nara M, Sugiyama Y, Sakanaka M, Yachi H, Kitakata A, Nakagawa A, Minami H, Okuda S, Katoh T, Katayama T, Kurihara S. 2017. Use of Gifu anaerobic medium for culturing 32 dominant species of human gut microbes and its evaluation based on short-chain fatty acids fermentation profiles. Biosci Biotechnol Biochem 81:2009–2017. doi:10.1080/09168451.2017.1359486. 28782454

[5] Koren S, Walenz BP, Berlin K, Miller JR, Bergman NH, Phillippy AM. 2017. Canu: scalable and accurate long-read assembly via adaptive k-mer weighting and repeat separation. Genome Res 27:722–736. doi:10.1101/gr.215087.116. 28298431 PMC5411767

[6] Walker BJ, Abeel T, Shea T, Priest M, Abouelliel A, Sakthikumar S, Cuomo CA, Zeng Q, Wortman J, Young SK, Earl AM. 2014. Pilon: an integrated tool for comprehensive microbial variant detection and genome assembly improvement. PLoS One 9:e112963. doi:10.1371/journal.pone.0112963. 25409509 PMC4237348

[7] Danylec N, Stoll DA, Dötsch A, Huch M. 2019. Draft genome sequences of type strains of *Gordonibacter faecihominis*, *Paraeggerthella hongkongensis*, *Parvibacter caecicola*, *Slackia equolifaciens*, *Slackia faecicanis*, and *Slackia isoflavoniconvertens*. Microbial Resour Announc 8:e01532-18. doi:10.1128/MRA.01532-18. PMC6328674 30643901

[8] Sayers EW, Agarwala R, Bolton EE, Brister JR, Canese K, Clark K, Connor R, Fiorini N, Funk K, Hefferon T, Holmes JB, Kim S, Kimchi A, Kitts PA, Lathrop S, Lu Z, Madden TL, Marchler-Bauer A, Phan L, Schneider VA, Schoch CL, Pruitt KD, Ostell J. 2019. Database resources of the National Center for Biotechnology Information. Nucleic Acids Res 47:D23–D28. doi:10.1093/nar/gky1069. 30395293 PMC6323993

[9] Li H, Durbin R. 2010. Fast and accurate long-read alignment with Burrows-Wheeler transform. Bioinformatics 26:589–595. doi:10.1093/bioinformatics/btp698. 20080505 PMC2828108

[10] Parks DH, Imelfort M, Skennerton CT, Hugenholtz P, Tyson GW. 2015. CheckM: assessing the quality of microbial genomes recovered from isolates, single cells, and metagenomes. Genome Res 25:1043–1055. doi:10.1101/gr.186072.114. 25977477 PMC4484387

[11] Tanizawa Y, Fujisawa T, Nakamura Y. 2018. DFAST: a flexible prokaryotic genome annotation pipeline for faster genome publication. Bioinformatics 34:1037–1039. doi:10.1093/bioinformatics/btx713. 29106469 PMC5860143

[12] Flórez AB, Vázquez L, Rodríguez J, Redruello B, Mayo B. 2019. Transcriptional regulation of the equol biosynthesis gene cluster in *Adlercreutzia equolifaciens* DSM19450^T^. Nutrients 11:993. doi:10.3390/nu11050993. PMC6566806 31052328

[13] Vázquez L, Flórez AB, Redruello B, Mayo B. 2020. Metabolism of soy isoflavones by intestinal bacteria: genome analysis of an *Adlercreutzia equolifaciens* strain that does not produce equol. Biomolecules 10:950. doi:10.3390/biom10060950. PMC7355428 32586036

[14] Toh H, Oshima K, Suzuki T, Hattori M, Morita H. 2013. Complete genome sequence of the equol-producing bacterium *Adlercreutzia equolifaciens* DSM 19450^T^. Genome Announc 1:e00742-13. doi:10.1128/genomeA.00742-13. 24051320 PMC3778203

