# Correction for Takahashi et al., “Complete Genome Sequence of Adlercreutzia equolifaciens subsp. *celatus* JCM 14811^T^”

**DOI:** 10.1128/MRA.00507-21

**Published:** 2021-06-03

**Authors:** Haruno Takahashi, Jiayue Yang, Hiromitsu Yamamoto, Shinji Fukuda, Kazuharu Arakawa

**Affiliations:** a Institute for Advanced Biosciences, Keio University, Tsuruoka, Yamagata, Japan; b Systems Biology Program, Graduate School of Media and Governance, Keio University, Fujisawa, Kanagawa, Japan; c Intestinal Microbiota Project, Kanagawa Institute of Industrial Science and Technology, Kawasaki, Kanagawa, Japan; d Transborder Medical Research Center, University of Tsukuba, Tsukuba, Ibaraki, Japan; e Faculty of Environment and Information Studies, Keio University, Fujisawa, Kanagawa, Japan; f Exploratory Research Center on Life and Living Systems, National Institutes of Natural Sciences, Myodaiji, Okazaki, Japan

## AUTHOR CORRECTION

Volume 10, no. 19, e00354-21, 2021, https://doi.org/10.1128/MRA.00354-21.

The title should read as given in this correction, and DSM 18785 should be changed to JCM 14811^T^ throughout. We primarily referred to the strain using the DSM strain number. However, the strain was obtained through RIKEN BRC, and RIKEN BRC requires the use of their JCM strain number. We apologize for the inconvenience caused.

